# Voltage dependence of synaptic plasticity is essential for rate based learning with short stimuli

**DOI:** 10.1038/s41598-018-22781-0

**Published:** 2018-03-15

**Authors:** Felix Weissenberger, Marcelo Matheus Gauy, Johannes Lengler, Florian Meier, Angelika Steger

**Affiliations:** Institute of Theoretical Computer Science, Department of Computer Science, ETHZ, 8092 Zürich, Switzerland

## Abstract

In computational neuroscience, synaptic plasticity rules are often formulated in terms of firing rates. The predominant description of *in vivo* neuronal activity, however, is the instantaneous rate (or spiking probability). In this article we resolve this discrepancy by showing that fluctuations of the membrane potential carry enough information to permit a precise estimate of the instantaneous rate in balanced networks. As a consequence, we find that rate based plasticity rules are not restricted to neuronal activity that is stable for hundreds of milliseconds to seconds, but can be carried over to situations in which it changes every few milliseconds. We illustrate this, by showing that a voltage-dependent realization of the classical BCM rule achieves input selectivity, even if stimulus duration is reduced to a few milliseconds each.

## Introduction

The ultimate goal of computational neuroscience is to understand the capabilities of the nervous system to represent and process information^1^. It is generally agreed that plastic synapses play a key role in the biophysical foundation of complex information processing. How plastic synapses change their efficacy as a function of the activity and state of presynaptic and postsynaptic neurons has been studied in numerous experiments. Based on these results, computational neuroscience aims to derive models of synaptic plasticity that admit to study what kind of computations may emerge in neuronal networks with plastic synapses.

Over the last decades there has been tremendous success in this endeavor, largely unburdened by using a *firing rate* abstraction of neuronal activity^2^. The accessibility of such *rate models* can be largely contributed to the fact that they permit an analysis for which one can resort to a large body of established mathematical tools^2,3^. A classic example is the Bienenstock Cooper Munro (BCM) theory, which reproduces the development of receptive fields in visual cortex^4^. More recent work focused on *spiking models* and demonstrated that plasticity rules formulated in terms of spike timing (STDP rules, e.g.^5–7^) and additionally in terms of the postsynaptic voltage (VDP rules, e.g.^8–11^) can be reduced to plasticity rules formulated in terms of firing rates (rate based plasticity rules, e.g.^4,12^) under the assumption that firing rates are a meaningful abstraction of neuronal activity^6,7,13^. As a consequence, current spiking network models, which are capable of remarkable computation, are often implementations of rate models with spiking neurons^14,15^. Whether rate or spiking models are suitable to describe neural computation in general and synaptic plasticity in particular is still highly debated^16–19^, see^20^ for review.

A critical limitation of all these models is that they rely on the assumption that firing rates encode the information that is relevant to perform the desired computation. However, a firing rate is a temporal average of spikes. For cortical neurons, which spike in a dynamic range of 0–200 Hz this average must be taken over milliseconds to seconds, as otherwise no spikes are observed and the concept of a firing rate is hollow^21^. This implies that rate based computation is restricted to computational tasks where information is encoded in slowly changing neuronal activity^3,17^.

This is in sharp contrast to the activity of cortical neurons in response to natural stimuli, which is typically characterized by the *instantaneous rate* (or firing probability) of the neuron. The instantaneous rate is reported in a peri-stimulus-time histogram (PSTH), which averages neuronal spiking over several repetitions of the same stimulus^3^. *In vivo* recordings of the instantaneous rate of cortical neurons in response to natural stimuli reveal that the activity of such neurons changes quickly, in the order of few milliseconds^17,22^. This suggests that for many computational tasks the relevant information is encoded in rapidly changing neuronal activity and thus a firing rate abstraction neglects a large amount of information.

It is currently unknown if and how the information encoded in the instantaneous rate is available to local synaptic plasticity mechanisms. The reason is that the instantaneous rate is an abstract concept whose computation requires several repetitions of identical stimuli, which in a natural environment are sparse, irregular and distant in time. In contrast, information encoded in the firing rate is directly accessible to local synaptic plasticity mechanisms via spikes, and the dependence of plasticity on firing rate^23–26^ and spike timing^26–30^ is well established.

In this work we resolve this discrepancy: we show that the instantaneous rate can be precisely estimated from the fluctuations of the membrane potential in *balanced networks*. Hence, the instantaneous rate is directly accessible to voltage-dependent synaptic plasticity mechanisms^31–33^. In balanced networks, excitatory inputs are canceled by inhibitory inputs on average^34–36^ and it is likely that cortical circuits operate in this balanced regime^37–39^.

Our result immediately implies that rate based plasticity rules that are linear in the presynaptic rate can be understood in terms of the instantaneous rate. Therefore, known insights on rate based plasticity transfer naturally to scenarios where relevant information is encoded in rapidly changing neuronal activity. So far, learning in such scenarios was only known to be feasible with STDP rules under the assumption of information being encoded in precise spike timing, in contrast to the rate based setup we study here.

Concretely, we analytically quantify how long neuronal activity, which encodes a certain stimulus in firing rate or instantaneous rate, must be stationary such that a plasticity rule can apply a desired weight change, which is given by an arbitrary function of the presynaptic and postsynaptic rates, with a given accuracy. Here we compare plasticity mechanisms that either solely depend on spiking of presynaptic and postsynaptic neurons (spike-dependent plasticity (SDP) rule, equivalent to STDP in a rate based setting) or additionally on the postsynaptic membrane potential (voltage-dependent plasticity (VDP) rule). We find that for fixed accuracy the neuronal activity may change at least one order of magnitude faster in the case of VDP compared to SDP, since VDP can utilize the instantaneous rate. We illustrate this on the example of the BCM rule to perform input selectivity of stimuli presented for a very short period of time (10 ms).

## Materials and Methods

### Neuron model

We use the classical model of Stein^40^ for cortical *in vivo* neuronal dynamics and its diffusion approximation^41^ (see^3,42^ for excellent introductions and^43^ for review). In Stein’s model a leaky integrate-and-fire (LIF) neuron is driven by stochastic spike arrival. The membrane potential $u(t)$ evolves according to

1 $$\tau \frac{{\rm{d}}}{{\rm{d}}t}u(t)=-u(t)+\tau \sum _{k=1}^{N}\sum _{{t}_{k}^{f}}{w}_{k}\delta (t-{t}_{k}^{f}),$$

where $\tau$ is the membrane time constant, $k$ indexes the $N$ synapses, ${t}_{k}^{f}$ are the spike arrival times, ${w}_{k}$ is the weight of the $k$-th synapse, and $\delta$ is the Dirac $\delta$-function. If the membrane potential reaches the threshold $\vartheta$, the neuron spikes and the membrane potential is set to the reset potential ${u}_{r}$ immediately afterwards. Hence, the action potential is not explicitly modeled.

The spikes arriving at the $k$-th synapse are generated by a Poisson process with rate ${\nu }_{k}$. The weights ${w}_{k}$ can be positive or negative corresponding to excitatory or inhibitory synapses respectively. We assume loosely balanced excitation and inhibition (see^44^ for review and Discussion): the mean ${\sum }_{k\mathrm{=1}}^{N}{w}_{k}{\nu }_{k}$ of the synaptic input is zero^34^. Hence, the rate $r$ of the neuron is determined by the variance of the synaptic input ${\sum }_{k\mathrm{=1}}^{N}{w}_{k}^{2}{\nu }_{k}$. This dependence is indicated by denoting the rate as $r(\sigma )$ for $\sigma \,:=\sqrt{\tau {\sum }_{k=1}^{N}{w}_{k}^{2}{\nu }_{k}}$.

For analytic tractability we consider the diffusion approximation of Stein’s model (where $N$ is large and ${w}_{k}$ small, also known as the synaptic bombardment assumption or high input regime^22^), which describes the membrane potential $u(t)$ as an Ornstein-Uhlenbeck process (OUP)

2 $${\rm{d}}u(t)=\frac{-u(t){\rm{d}}t}{\tau }+\frac{\sigma }{\sqrt{\tau }}{\rm{d}}{W}_{t},$$

where ${\rm{d}}{W}_{t}$ are the increments of a Wiener process in time ${\rm{d}}t$^41^. The spike generation at threshold $\vartheta$ followed by a reset to ${u}_{r}$ is analogous to Stein’s model. The diffusion approximation allows to determine the rate as the inverse of the expected first passage time of the OUP given by Siegert’s formula^42^ as

3 $$r(\sigma )={(\tau \sqrt{\pi }{\int }_{\frac{{u}_{r}}{\sigma }}^{\frac{\vartheta }{\sigma }}\exp ({x}^{2})\cdot (1+{\rm{e}}{\rm{r}}{\rm{f}}(x)){\rm{d}}x)}^{-1},$$

where erf denotes the error function. Furthermore, as a consequence of balanced excitation and inhibition the neuron operates in the fluctuation-driven regime and its interspike interval (ISI) distribution is exponential (in the limit of large $\vartheta /\sigma$). Hence, the neuron spikes according to a Poisson process with rate $r(\sigma )$^22,45^. As a consequence, in a time interval during which $\sigma$ is constant, $r(\sigma )$ describes both the firing rate and the instantaneous rate of the neuron. Therefore, in the sequel we will simply continue referring to it as rate, and the length of the considered time interval indicates whether it makes sense to think of it as the firing rate (time intervals in the order of seconds) or instantaneous rate (time intervals in the order of milliseconds).

### Information about the stimulus

Learning with local plasticity rules is limited by the amount of information about the stimulus, encoded in the neuronal activity of presynaptic and postsynaptic neurons, available per time. This amount of information, termed Fisher information, is quantified as the inverse variance of an *optimal estimator* (an estimator with minimal variance among all estimators) of the stimulus (see^46^ for an introduction). In this section we analytically compare two local neuronal observables, namely spike count and membrane potential, with respect to how much information they convey about the stimulus, which is encoded in a rate. Concretely, we compute the variance of optimal rate estimators based on either spike times or voltage samples.

#### Information from spiking

As a consequence of balanced excitation and inhibition, the neuron spikes according to a Poisson process with rate $r$. Let ${t}_{1},\ldots ,{t}_{n}$ be the spike times observed in a time interval of length $T$. The maximum likelihood estimator of the rate of a Poisson process, which is an optimal estimator of the rate, is given by

4 $${\hat{r}}^{{\rm{spike}}}=\frac{n}{T},$$

and has variance

5 $${{\rm{Var}}}_{r}^{{\rm{spike}}}=\frac{r}{T},$$

see^46^. Hence, the Fisher information of the rate in a Poisson spike train is proportional to the length of the observed time interval. Further, as a consequence of the Poisson model, the actual spike times are irrelevant. Therefore, if the rate estimate is based solely on spiking, then one can only increase the amount of information about the rate by observing the neuron for a longer time interval.

#### Information from membrane potential

The membrane potential evolves according to an OUP as in Equation (2) and the neuron spikes with rate $r(\sigma )$, see Equation (3). Observing the membrane potential to extract information is modeled by taking samples ${\bf{u}}:={u}_{0},\ldots ,{u}_{n}$ of the membrane potential in a time interval of length $T$. Possible postsynaptic action potentials are not contained in the membrane potential trajectory as they are not explicit in the LIF neuron model. We assume equidistant sampling times with distance $\varepsilon \,:=T/n$ and refer to $\mathrm{1/}\varepsilon$ as the sampling rate. The transition probability density of the OUP is

$$p(u,t| u^{\prime} ,t^{\prime} )=\frac{1}{\sqrt{\pi {\sigma }^{2}(1-\exp (-{\textstyle \tfrac{2(t-t^{\prime} )}{\tau }}))}}\cdot \exp (-\frac{{(u-u^{\prime} \exp (-{\textstyle \tfrac{t-t^{\prime} }{\tau }}))}^{2}}{{\sigma }^{2}(1-\exp (-{\textstyle \tfrac{2(t-t^{\prime} )}{\tau }}))}),$$

see for example^42^. This is the probability that $u(t)$ is equal to $u$ given that $u(t^{\prime} )$ was equal to $u^{\prime}$. Therefore, the likelihood of the samples is

$$L(\sigma ;{\bf{u}})=\prod _{i=0}^{n-1}\frac{1}{\sqrt{\pi {\sigma }^{2}(1-\exp (-{\textstyle \tfrac{2\varepsilon }{\tau }}))}}\cdot \exp (-\frac{{({u}_{i+1}-{u}_{i}\exp (-{\textstyle \tfrac{\varepsilon }{\tau }}))}^{2}}{{\sigma }^{2}(1-\exp (-{\textstyle \tfrac{2\varepsilon }{\tau }}))}),$$

the log-likelihood (only terms depending on $\sigma$ are shown) is

$$l(\sigma ;{\bf{u}})=\ldots -n\mathrm{log}\sigma -\frac{1}{{\sigma }^{2}}\sum _{i=0}^{n-1}\frac{{({u}_{i+1}-{u}_{i}\exp (-{\textstyle \tfrac{\varepsilon }{\tau }}))}^{2}}{(1-\exp (-{\textstyle \tfrac{2\varepsilon }{\tau }}))},$$

and the first derivative of the log-likelihood with respect to $\sigma$ is

$$\frac{\text{d}}{\text{d}\sigma }l(\sigma ;{\bf{u}})=-\frac{n}{\sigma }+\frac{2}{{\sigma }^{3}}\sum _{i=0}^{n-1}\frac{{({u}_{i+1}-{u}_{i}\exp (-{\textstyle \tfrac{\varepsilon }{\tau }}))}^{2}}{(1-\exp (-{\textstyle \tfrac{2\varepsilon }{\tau }}))}.$$

Thus, by the invariance principle^46^, an optimal estimator of the rate is then given by

6 $${\hat{r}}^{{\rm{v}}{\rm{o}}{\rm{l}}{\rm{t}}{\rm{a}}{\rm{g}}{\rm{e}}}=r(\hat{\sigma }),$$

with

7 $$\hat{\sigma }=\sqrt{\frac{2\sum _{i=0}^{n-1}{({u}_{i+1}-{u}_{i}\exp (-\tfrac{\varepsilon }{\tau }))}^{2}}{n\cdot \mathrm{(1}-\exp \,(-\tfrac{2\varepsilon }{\tau }))}}{\rm{.}}$$

The expectation of the second derivative of the log-likelihood with respect to $\sigma$ is

$$\frac{{{\rm{d}}}^{2}}{{\rm{d}}{\sigma }^{2}}l(\sigma ;{\bf{u}})=-\frac{2T}{{\sigma }^{2}\varepsilon }{\rm{.}}$$

Therefore, the variance of $\hat{\sigma }$ is ${\sigma }^{2}\varepsilon \mathrm{/(2}T)$. Using the invariance principle and the delta method^46^ we conclude that the variance of the rate estimator is

8 $${{\rm{Var}}}_{r}^{{\rm{voltage}}}=\frac{{\sigma }^{2}\varepsilon }{2T}\cdot {(\frac{{\rm{d}}}{{\rm{d}}\sigma }r(\sigma ))}^{2}{\rm{.}}$$

Note that the amount of information about the rate extractable from the membrane potential is not only proportional to the duration of observation but crucially also to the sampling rate. Therefore, if the rate estimate is based on the membrane potential, then the amount of information about the rate can be increased by a higher sampling rate. However, the sampling rate must be smaller than the spike arrival rate, which led to the approximation of the membrane potential by an OUP, as otherwise this approximation is not valid, see Discussion.

#### Time improvement

Let ${T}^{{\rm{spike}}}$ and ${T}^{{\rm{voltage}}}$ be the duration of a stimulus that is required to extract a certain amount of information about the stimulus either from the spike train or the membrane potential evolution of a neuron encoding it. The factor of time improvement is given by

9 $$\frac{{T}^{{\rm{spike}}}}{{T}^{{\rm{voltage}}}}=\frac{2{r}_{{\rm{post}}}}{{\sigma }^{2}\varepsilon {(\frac{{\rm{d}}}{{\rm{d}}\sigma }r(\sigma ))}^{2}},$$

combining Equations (5) and (8).

### Rate based plasticity with spiking neurons

A rate based plasticity rule describes the synaptic weight change ${{\rm{\Delta }}}_{w}:=f({r}_{{\rm{pre}}},\,{r}_{{\rm{post}}})$ as a function of the presynaptic and postsynaptic rates ${r}_{{\rm{pre}}}$ and ${r}_{{\rm{post}}}$. A general plasticity rule *realizes* a particular rate based rule $f$ if the expected weight change of the synaptic weight is equal to ${{\rm{\Delta }}}_{w}$, after the presynaptic and postsynaptic neurons spiked with rates ${r}_{{\rm{pre}}}$ and ${r}_{{\rm{post}}}$ for time $T$ (the expectation is over the randomness of the Poisson spike trains)^6,7,13^. Crucially for learning, the actual weight change should be close to its expectation. Hence, an *optimal plasticity rule* minimizes the variance of the weight change, among all rules applying the same expected weight change.

#### Optimal SDP rule

We now derive a lower bound for the variance of the weight change ${{\rm{Var}}}_{w}^{{\rm{spike}}}$, induced by the postsynaptic variability, of any SDP rule realizing $f$. Applying a SDP rule for time $T$ can be seen as a protocol to estimate the weight change ${{\rm{\Delta }}}_{w}$. Hence, by the invariance principle we can obtain an optimal estimator for ${{\rm{\Delta }}}_{w}$ as

10 $${\hat{{\rm{\Delta }}}}_{w}^{{\rm{spike}}}=f({\hat{r}}_{{\rm{pre}}}^{{\rm{spike}}},{\hat{r}}_{{\rm{post}}}^{{\rm{spike}}}),$$

where ${\hat{r}}_{{\rm{pre}}}$ and ${\hat{r}}_{{\rm{post}}}$ are optimal estimators for the presynaptic and postsynaptic rates, given in Equation (4). This immediately defines an optimal SDP realization of $f$: first, estimate the rates according to Equation (4) and thereafter apply $f$ to the estimates (the optimal voltage based rule is analogous, using Equation (6) respectively). By the delta method and Equation (5) we derive that the variance of the optimal estimator ${\hat{{\rm{\Delta }}}}_{w}^{{\rm{spike}}}$ is

11 $${{\rm{Var}}}_{w}^{{\rm{spike}}}=\frac{{r}_{{\rm{post}}}}{T}\cdot {(\frac{\partial }{\partial {r}_{{\rm{post}}}}f({r}_{{\rm{pre}}},{r}_{{\rm{post}}}))}^{2}{\rm{.}}$$

Thus, we can conclude that each SDP rule applies a weight change with variance at least the variance computed above.

#### Optimal VDP rule

Let ${{\rm{Var}}}_{w}^{{\rm{voltage}}}$ be the variance of the weight change induced by the postsynaptic variability of an optimal VDP rule. Analogously to the derivation of Equation (10), the optimal VDP realization is given by

12 $${\hat{{\rm{\Delta }}}}_{w}^{{\rm{voltage}}}=T\cdot f({\hat{r}}_{{\rm{pre}}}^{{\rm{spike}}},{\hat{r}}_{{\rm{post}}}^{{\rm{voltage}}}),$$

according to the invariance principle, and using the delta method together with Equation (8) we conclude

13 $${{\rm{Var}}}_{w}^{{\rm{voltage}}}=\frac{{\sigma }^{2}\varepsilon }{2T}\cdot {(\frac{{\rm{d}}}{{\rm{d}}\sigma }r(\sigma ))}^{2}\cdot {(\frac{\partial }{\partial {r}_{{\rm{post}}}}f({r}_{{\rm{pre}}},{r}_{{\rm{post}}}))}^{2}{\rm{.}}$$

#### Time scale of stimuli

Combining Equations (11) and (13) immediately shows that for fixed variance the relative improvement factor of required stimulus duration for learning is given by Equation (9). This factor determines how much longer a stimulus needs to be stationary in case of SDP compared to VDP to achieve the same accuracy in the desired weight change. In particular, this relative improvement factor is independent of the plasticity rule $f$, thus allows to conclude a general advantage of VDP over SDP regarding the time scale in which stimuli must be stationary.

### Selectivity with the BCM rule

The BCM theory^4^ is one of the most influential rate based learning theories (see^47^ for a review). The BCM rule maximizes selectivity and can reproduce formation of receptive fields in the visual cortex^47^. In this section we derive optimal SDP and VDP realizations of the BCM rule and define the computational task of selectivity. This task will later serve as an example of how to transform a rate based computational task into a fast spiking model.

#### BCM rule

The BCM rule defines the change in synaptic weight as $f({r}_{{\rm{pre}}},\,{r}_{{\rm{post}}})={r}_{{\rm{pre}}}\cdot \varphi ({r}_{{\rm{post}}},\,{\overline{r}}_{{\rm{post}}})$, with nonlinear function $\varphi$ and postsynaptic reference rate ${\overline{r}}_{{\rm{post}}}$. The function $\varphi$ displays long-term depression (LTD) for low postsynaptic rate and long-term potentiation (LTP) for high postsynaptic rate, see Fig. 2(a). Further, ${\overline{r}}_{{\rm{post}}}$ determines a sliding threshold between LTD and LTP, which depends nonlinearly on ${r}_{{\rm{post}}}$ on a slower time scale, and increases (decreases) if ${r}_{{\rm{post}}}$ has been large (small) for some time.

#### Optimal SDP and VDP realizations of the BCM rule

Let us formally define the BCM rule, with a particular choice of $\varphi$ and sliding threshold, following^48^, and its optimal SDP and VDP realizations. The weight change in a short time interval of length $T$ during which the rates are assumed to be constant is

14 $${{\rm{\Delta }}}_{w}=\eta \cdot {r}_{{\rm{pre}}}\cdot ({r}_{{\rm{post}}}^{2}-{\overline{r}}_{{\rm{post}}}\cdot {r}_{{\rm{post}}}),$$

where $\eta  > 0$ is the step size. The change of the sliding threshold ${\overline{r}}_{{\rm{post}}}$ is defined by

15 $${\rm{\Delta }}{\overline{r}}_{{\rm{post}}}=\frac{({r}_{{\rm{post}}}^{2}-{\overline{r}}_{{\rm{post}}})}{{\tau }_{{\rm{BCM}}}},$$

with time constant ${\tau }_{{\rm{BCM}}}$.

We now introduce optimal realizations, which achieve minimum variance among SDP^7,13^ and VDP^8–11^ realizations of the BCM rule. Since the BCM rule is linear in the presynaptic rate, both SDP and VDP realizations simply perform a weight update for each presynaptic spike. Assume that in a short time interval of length $T$, the presynaptic and postsynaptic cells spike with constant rate. According to Equation (10) and Equation (4), the optimal weight update of a SDP rule is then given by

16 $$\eta \cdot ({(\frac{{n}_{{\rm{post}}}}{T})}^{2}-{\overline{r}}_{{\rm{post}}}\cdot \frac{{n}_{{\rm{post}}}}{T}),$$

where ${n}_{{\rm{post}}}$ is the number of postsynaptic spikes in the interval.

According to Equation (12) and Equation (6), the optimal weight update of a VDP rule is given by

17 $$\eta \cdot (r{(\hat{\sigma })}^{2}-{\overline{r}}_{{\rm{post}}}\cdot r(\hat{\sigma })),$$

with $\hat{\sigma }$ as in Equation (7). The implementation of the sliding threshold in Equation (15) is analogous. The resulting VDP realization of the BCM rule relates fluctuations in the membrane potential to a synaptic weight change. This is in contrast to previous VDP rules, which have been shown to realize BCM under the assumption of Poisson spike trains, since they rely on low-pass filtered versions of the membrane potential and thus cannot exploit the information in the fluctuations^8–11^.

#### Selectivity

The task of selectivity is that a neuron becomes selective to one particular stimulus out of a set of stimuli. Here we formulate this task (based on the simulation paradigm of^49^) in a spiking model.

We consider a feed forward network with $N$ excitatory input neurons and one output neuron, see Fig. 2(b) and (f). A stimulus is described by ${\boldsymbol{\nu }}=({\nu }_{1},\ldots ,{\nu }_{N}{)}^{{\rm T}}$, where the $k$-th component corresponds to the rate of the $k$-th input neuron. Moreover, we denote the vector of synaptic weights by ${\bf{w}}=({w}_{1},\ldots ,{w}_{N}{)}^{{\rm{T}}}$, hence the weight of the synapse connecting the $k$-th excitatory neuron with the output neuron is ${w}_{k}$. To model balanced excitation and inhibition, each excitatory input neuron is accompanied by an inhibitory neuron and the respective weights are mirrored. It has been shown in^36^ how this mirroring is achieved by inhibitory plasticity in an experience dependent manner. Thus, the expected input to the output neuron is zero and the variance of the input is $2{\sum }_{k\mathrm{=1}}^{N}{\nu }_{k}{w}_{k}^{2}$. Consequently, the rate of the output neuron is $r(\sigma )$ with $\sigma \,:=\sqrt{2\tau {\sum }_{k=1}^{N}{w}_{k}^{2}{\nu }_{k}}$. We stress that $\sigma$ is a function of the stimulus and the weight, but do not indicate this in the notation for simplicity.

Let ${\{{\nu }^{(j)}\}}_{j\mathrm{=1}}^{m}$ denote a set of $m$ stimuli and let ${\sigma }^{(j)}\,:=\sqrt{2\tau {\sum }_{k=1}^{N}{w}_{k}^{2}{\nu }_{k}^{(j)}}$. Moreover, let ${p}_{j}$ be the probability of the $j$-th stimulus (the ${p}_{j}$’s form a probability distribution $P$ over stimuli). The selectivity of the output neuron for a given weight vector ${\bf{w}}$ is defined as

18 $${\rm{Sel}}({\bf{w}}\mathrm{)=1}-\frac{{{\mathbb{E}}}_{P}[r(\sigma )]}{{{\rm{\max }}}_{j}\,r({\sigma }^{(j)})}{\rm{.}}$$

Further, we round down responses below 1 Hz to 0 Hz and apply the convention that $\mathrm{0/0}=1$ to avoid trivial selectivity. Note that if all stimuli result in the same postsynaptic response, then the selectivity is $0$, however if the response is nonzero for exactly one stimulus and zero for all others, the selectivity is at its maximum $1-1/m$.

#### Simulation protocol

The BCM rule makes the weights converge to a maximally selective fixed point. The simulation protocol is as follows: first, fix the duration of stimulus presentation $T$. Thereafter, in each round pick a stimulus ${\nu }^{(j)}$ according to the probability distribution $P$ and simulate the output neuron for time $T$ with the corresponding input. From this derive ${n}_{{\rm{post}}}$, the number of spikes of the neuron and ${u}_{1},\ldots ,{u}_{n}$, the samples of the membrane potential. Then update the weight of the $k$-th synapse according to Equation (16), for the SDP rule, and Equation (17), for the VDP rule, multiplied by ${\nu }_{k}^{(j)}$. After each round, the sliding threshold is updated according to Equation (15), using the respective estimators of the postsynaptic rate.

### Data availability

**Figure 1:**
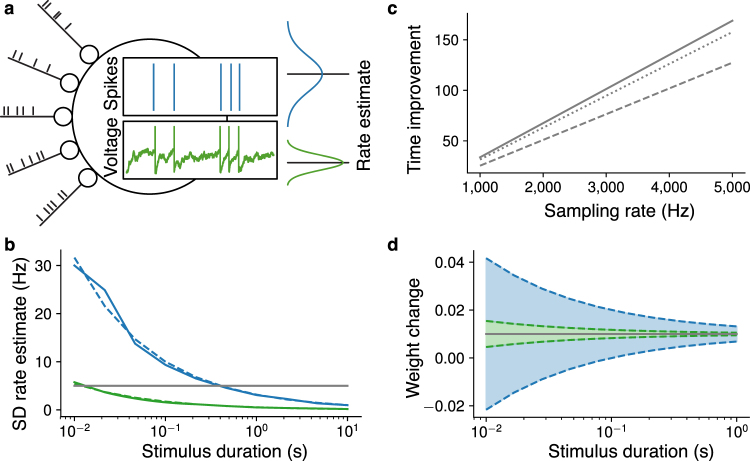
Required stimulus duration of SDP and VDP rules. (**a**) Obtaining information about the rate from spikes (blue) and voltage (green). The amount of information is quantified as the inverse variance of the optimal rate estimate (Fisher information). (**b**) Standard deviation (SD) of the rate estimate based on spikes (blue) and voltage (green) as function of stimulus duration. Horizontal grey line indicates that for fixed information level the required duration differs by an order of magnitude. Dashed lines correspond to Equations (5) and (8), solid lines are respective simulations (empirical SD of estimates according to Equations (4) and (6) of a simulated neuron). (**c**) Factor of time improvement for information extraction as a function of sampling rate according to Equation (9), for different firing rates 10 Hz (solid), 20 Hz (dotted), 40 Hz (dashed). (**d**) Weight change as function of stimulus duration. Grey horizontal line indicates desired weight change, shaded areas show one SD of the weight change applied by optimal SDP rule (blue) and VDP rule (green) according to Equations (11) and (13). Parameters (if not varied in the respective plot) are $r=10\,{\rm{Hz}}$, $\vartheta =-55\,{\rm{mV}}$, ${u}_{r}=-70\,{\rm{mV}}$, $\tau =0.02\,{\rm{s}}$, $1/\varepsilon =1\,{\rm{kHz}}$, 100 trials.

**Figure 2:**
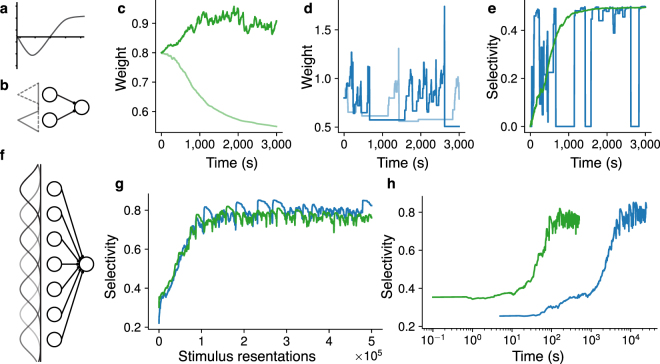
Fast selectivity with BCM and natural stimuli. (**a**) The BCM learning rule; weight change as function of the postsynaptic rate. (**b**) Task with orthogonal stimuli (dashed gray and gray) and two input neurons; for orthogonal stimuli the weights converge to a maximally selective fixed point (rate based analysis). (**c**) Evolution of the two weights (light and dark green) from (**b**) over time for the optimal VDP realization of BCM. (**d**) Evolution of the weights (light and dark blue) from (**b**) over time for the optimal SDP realization of BCM. (**e**) Respective selectivity of the weights in (**c**) and (**d**) over time; while the VDP rule (green) converges, the SDP (blue) jumps out of the maximally selective fixed point. Parameters in (**c**), (**d**) and (**e**) are $N=2$, $m=2$, peak rate $10\,{\rm{Hz}}$, initial weights $0.8\,{\rm{mV}}$, $T=10\,{\rm{ms}}$, $\vartheta =-55\,{\rm{mV}}$, ${u}_{r}=-70\,{\rm{mV}}$, $\tau =0.02\,{\rm{s}}$, $1/\varepsilon =1\,{\rm{kHz}}$, $\eta =0.000001$, ${\tau }_{BCM}=1000$, $\theta =1\,{\rm{Hz}}$. (**f**) Task with non-orthogonal stimuli (Gaussian rate profiles). For such stimuli, BCM still increases the selectivity (rate based simulation). (**g**) Selectivity as a function of the number of stimulus presentations; duration of individual stimuli is 500 ms for SDP rule (blue) and 10 ms for the VDP rule. (**h**) Selectivity as a function of time (log scale). Duration of stimuli is chosen such that the variance of the weight change for SDP rule (blue) and VDP rule (green) match and are small to allow close to optimal selectivity, see (**g**). Parameters in (**g**) and (**h**) are $N=100$, $m=10$, peak rate 10 Hz, base rate 2 Hz, standard deviation of Gaussian rate profile 10, initial weights 0.1 mV, $\vartheta =-55\,{\rm{mV}}$, ${u}_{r}=-70\,{\rm{mV}}$, $\tau =0.02\,{\rm{s}}$, $1/\varepsilon =1\,{\rm{kHz}}$, $\eta =0.000001$, ${\tau }_{BCM}=1000$, $\theta =1\,{\rm{Hz}}$.

The data shown in Figs 1 and 2 is obtained from simulations that can be reproduced given the simulation code, see code availability below.

### Code availability

All simulations are performed using the python programming language and the source-code is available on request.

## Results

Our first result shows that the information about the stimulus extractable per time from the membrane potential is much higher than the information content of the spike train, see Fig. 1(a) and Materials and methods. This is a consequence of excitation and inhibition being balanced, because in the balanced setting, the membrane potential changes due to a large number of input spikes, while the neuron produces only few output spikes^22^, see also Discussion. The Fisher information about the rate, which encodes stimuli, obtained from neuronal spiking is proportional to the duration of observation, see Equation (5). Thus, if the stimulus is present only for short time, then only a limited amount of information about it is available to a synaptic plasticity mechanism depending solely on spiking. The Fisher information obtained from the membrane potential is not only proportional to the duration of observation, but also to the sampling rate, see Equation (8). Hence, if the stimulus is present only for short time, the amount of information extractable from the membrane potential can exceed the limit of the spike based case. We illustrate this with a concrete example: consider the neuron firing with 10 Hz. How long does it take to obtain an estimate of the rate that is within 5 Hz accuracy with 70% confidence? Based on neuronal spiking this takes at least 500 ms, however, sampling the membrane potential with a sampling rate of 1 kHz requires only 10 ms, see Fig. 1(b) grey line. Therefore, in the latter it is fine if the stimulus changes every 10 ms. This is in the order in which the instantaneous rate changes *in vivo*^22^. Thus, it is possible to extract information about the instantaneous rate from voltage traces in contrast to spike trains of the same duration. The relative time improvement of voltage based estimation over spike based, given in Equation (9), is at least one order of magnitude for typical neuronal parameters, see Fig. 1(c).

Our second result directly relates the previous observation to synaptic plasticity. A rate based plasticity rule defines the synaptic weight change as a function of the presynaptic and postsynaptic firing rates. We derive optimal SDP and VDP realizations of any rate based rule in Materials and methods. For optimal SDP rules, the variance of the applied weight change scales as the inverse of the Fisher information about the rate obtained from neuronal spiking, see Equation (11), whereas for optimal VDP rules the variance scales as the inverse of the Fisher information obtained from the membrane potential, see Equation (13). In particular, if a stimulus is stationary only on a short time scale, the SDP rule applies a weight change that can be far from the desired weight change. In contrast, a VDP rule can still be highly accurate, see Fig. 1(d). This “speed” improvement of VDP rules over SDP rules is determined in Materials and methods and is equal to the improvement of information retrieval, see Equation (9). Hence, the improvement factor is independent of the specific learning rule at hand. Thus, this constitutes a general improvement in learning speed for VDP over SDP and highlights that VDP can operate on the timescale in which the instantaneous rate changes *in vivo*.

Finally, we illustrate the previous considerations on the classic learning task of selectivity, see Materials and methods. Given a collection of stimuli in the form of rate profiles of input neurons (e.g., representing the activity of the lateral geniculate nucleus (LGN) induced by an angular bar sweep), the task is to make the output neuron selective for one particular stimulus: the output neuron should strongly respond to one stimulus and remain quiet for any other stimulus. The network and the stimuli are depicted schematically in Fig. 2(b) and (f). This task is solved by the BCM learning rule acting on the synapses^4^.

First, we study two orthogonal stimuli presented to the network. In each stimulus one input neuron spikes with a certain rate while the other input neuron is quiet, see Fig. 2(b). For orthogonal stimuli the BCM rule guarantees that the weight vector converges to a maximally selective fixed point, if the stimuli are presented randomly, round by round, for a certain duration. We choose the same stimulus durations for both the VDP and the SDP rule (10 ms), which reflects the time scale on which the instantaneous rate changes *in vivo*^22^. For the optimal VDP realization of the BCM rule, given in Equation (17), the weights converge to a maximally selective fixed point, see Fig. 2(c). For the same stimulus duration, the variance of the weight changes induced by the optimal SDP realization of BCM, given in Equation (16), is much larger, see Fig. 2(d). This variability results in bad performance because the weights leave the maximally selective fixed point, causing instability, see Fig. 2(e). Therefore, to bound the variance of the weight change and thus guarantee stability, the stimuli must be available significantly longer for the SDP rule than for the VDP rule.

We next investigate the performance for more realistic stimuli^49^. Here, each stimulus has a Gaussian profile with certain peak and base rates and standard deviation, see Fig. 2(f). With such stimuli, the convergence is not guaranteed, and the maximal selectivity decreases with increasing base/peak rate ratio and standard deviation of the Gaussian profile. Since the weight vector does not converge, but BCM only increases the selectivity, the variance of the weight changes induced by learning determines how selective the neuron can be. We now choose different stimulus durations for the VDP rule (10 ms) and the SDP rule (500 ms). With significantly longer stimulus duration for the SDP rule, both realizations of BCM yield similar performance, shown as a function in the number of stimulus presentations in Fig. 2(g). This implies that the total exposition time of the neuron to stimuli is at least an order of magnitude smaller for the VDP rule compared to SDP rule, see Fig. 2(h) where the selectivity is shown as a function of total exposition time on a log scale.

## Discussion

We use Stein’s model and its diffusion approximation as an abstraction for cortical *in vivo* neuronal dynamics, following the influential paper by Michael N. Shadlen and William T. Newsome^22^. Their work, which contains a detailed discussion of the biological justifications^38^ and limitations of the model, points out a crucial property implied by excitation and inhibition being balanced: the neuron produces a highly variable spike train, which is essentially independent of the spike timing of the presynaptic neurons. This is consistent with experimental observations^16,50^. We approximate Stein’s model by its diffusion approximation. The diffusion approximation is justified by the large number of postsynaptic potentials (PSPs) arriving at cortical neurons, a phenomenon known as high input or synaptic bombardment regime. Rough estimates (100–1000 neurons out of 1000–10000 input neurons spike with a rate of 10 Hz) yield a spike arrival rate in the order of 1 kHz −10 kHz^22^. In our approach we estimate the variance of the diffusion approximation by sampling the membrane potential. It is clear that the sampling rate cannot be higher than the arrival rate because otherwise the approximation would be invalid (the difference between two samples is assumed be a Gaussian, but if the sampling rate is too high, then this assumption does not hold). Hence, the arrival rate determines a natural upper bound for a reasonable sampling rate. Notably, taking the diffusion approximation is not necessary to recover our results qualitatively: in Stein’s model (allowing for fewer and stronger synaptic inputs) the information about the rate contained in the membrane potential trajectory typically exceeds the information in spike trains as long as the arrival rate is significantly higher than the output rate.

Experiments revealed that synaptic plasticity depends on the presynaptic and postsynaptic rates^23–26^, the exact time difference of presynaptic and postsynaptic spikes^27–30^, the postsynaptic membrane potential^31–33^, and ultimately the calcium concentration in the postsynaptic dendritic spine in consequence of voltage-dependent calcium and N-methyl-D-aspartate receptor (NMDAR) channel activation^51,52^. Modest calcium levels cause LTD whereas high levels result in LTP^53,54^. Hence, via voltage-gated calcium and NMDAR channels, plasticity is inherently voltage dependent. Thus, the magnitude of voltage fluctuations may translate to different levels of calcium concentration. In particular, as the calcium influx depends nonlinearly on the voltage due to channel activation thresholds, larger voltage fluctuations might lead to a higher calcium concentration even if the mean voltage stays unchanged. This establishes a possible link between voltage fluctuations and plasticity. However, it is not clear if there exists a mechanism that implements the estimator in Equation (7) and thereby exploits a high sampling rate to estimate the voltage fluctuations precisely.

In our neuron model the somatic membrane potential is a local observable at the postsynaptic part of the synapse. However, this is a strong assumption, which is only legitimate for synapses close to the soma. For synapses on distant dendritic spines, the somatic membrane potential can be replaced by a local potential in the dendritic compartment, which potentially still contains more information about the postsynaptic instantaneous rate than single back propagating action potentials (BAPs)^55^.

Notably, our approach requires a biophysical pathway that transports information about the fluctuations of the somatic membrane potential of the postsynaptic neuron (and thus its instantaneous rate) back along the dendrites to the postsynaptic site of a synapse. There is experimental evidence that this is possible for the mean of the membrane potential^31–33^ and see^56^ for a review of the voltage dependence of LTP and LTD. This pathway does not need to be fast, and the signalling mechanism does not necessarily need to be a voltage signal.

It has been observed that excitatory and inhibitory synaptic inputs to cortical neurons exhibit strong temporal and quantitative relations, a phenomenon termed balanced excitation and inhibition, see^57^ for review. One distinguishes two types of balance: (1) *loose balance*, where a large number of uncorrelated small excitatory and inhibitory synaptic inputs cancel each other out on average (2) *tight balance*, where inhibition closely tracks excitation with a very short time lag, see^44^. Loose balance was postulated to explain the high-degree variability in neuronal responses to natural stimuli^16,22,37^. This led to a widely accepted class of network models (balanced networks) that display asynchronous irregular spiking dynamics^34,35,39^, resembling the activity in many cortical areas. Tight balance has been suggested to be a signature of highly efficient coding, see^44^ and the references therein, however it is not consistent with trial to trial variability of neuronal responses and asynchronous irregular firing^58^.

We model loose balance that is maintained over time and stimuli (termed detailed balance in^36^). Hence, the mean of the membrane potential $\mu$ is constant over time and stimuli. Therefore, stimuli are encoded in the fluctuations of the membrane potential, rather than its mean, see Equation (3). This implies that the instantaneous rate can be decoded from the membrane potential quickly depending on the sampling rate, see Equation (8). Without balance, we would have to write the rate as $r(\mu ,\sigma )$ and the variance of an optimal rate estimator based on the membrane potential becomes

19 $${{\rm{Var}}}_{r}^{{\rm{voltage}}}=\frac{{\sigma }^{2}\varepsilon }{2T}\cdot {(\frac{\partial }{\partial \sigma }r(\mu ,\sigma ))}^{2}+\frac{{\sigma }^{2}\tau }{T}\cdot {(\frac{\partial }{\partial \mu }r(\sigma ,\mu ))}^{2},$$

derived along the lines of Equation (8). In this case the information about the rate in the membrane potential cannot simply be increased by a higher sampling rate, since the variance of the mean potential only decreases with observation time $T$ and the membrane time constant $\tau$, indicated by the second term of Equation (19). It turns out that the time scale of information extraction is thus in the same order as in the spike based case. This reveals that one functional advantage of the loosely balanced state is efficient encoding of the instantaneous rate in the membrane potential.

The main prediction of our model is how synaptic plasticity depends on the variance of the postsynaptic membrane potential, assuming a specific rate based learning rule, for example the BCM rule. So far, voltage dependence has only been studied with fixed postsynaptic (super-threshold) depolarization, inconsistent with *in vivo* conditions, without controlling the variance of the depolarization^31–33^. This revealed the existence of a voltage threshold for LTD and a higher voltage threshold for LTP induction^31^. It would be interesting to study high variance depolarization because in this way both thresholds are reached and it is not clear how or if the LTP and LTD components are combined. Concretely, the rate of the postsynaptic neuron can be controlled in two ways by current injection: (1) by injecting a current with large mean and zero variance (2) by injecting a current with small mean and large variance^38^. Our hypothesis is that the effect on the synaptic efficacy only depends on the rate of the neuron, not how it is induced. If this does not hold true, this would give an argument against rate based plasticity models.

Furthermore, our model predicts that as a consequence of loosely balanced excitation and inhibition the instantaneous rate can be well estimated from voltage recordings. To test this hypothesis one can compute the instantaneous rate of a neuron *in vivo* using two protocols. The classic protocol is via construction of the PSTH from many spike train recordings. Our proposed protocol is to estimate it via Equation (6) from a single or few voltage recordings. We hypothesize that the number of required voltage recordings is much smaller than the number of spike train recordings in order to get a certain accuracy. As a consequence the number of required repetitions of the experiment can be reduced in order to compute the instantaneous rate and interestingly the instantaneous rate could also be computed in scenarios where the experiment cannot be repeated at all since the stimulus is actually unknown.
